# Comparison Between Laparoscopic and Open Right Hemicolectomy Outcomes: A Single-Centre Analysis

**DOI:** 10.3390/medicina62040655

**Published:** 2026-03-29

**Authors:** Vasiliki Garantzioti, Ioannis D. Kostakis, George Theofanis, Ioannis Maroulis, George Skroubis

**Affiliations:** Department of Surgery, University Hospital of Patras, Faculty of Medicine, University of Patras, 26504 Patras, Greece; vigarant@yahoo.com (V.G.); ioannis.kostakis@gmail.com (I.D.K.);

**Keywords:** laparoscopic right hemicolectomy, open right hemicolectomy, intracorporeal anastomosis, outcomes, colorectal cancer

## Abstract

*Background and Objectives*: Laparoscopic procedures have become a routine approach in colorectal surgery. We aimed to evaluate intraoperative, postoperative and pathological outcomes of laparoscopic right hemicolectomy in comparison with open right hemicolectomy. *Materials and Methods*: We reviewed our database for colorectal surgery and collected data regarding right hemicolectomies performed over a period of 10 years regarding patient characteristics, operative outcomes and postoperative outcomes. We compared laparoscopic with open right hemicolectomies. All the anastomoses in the laparoscopic group were performed intracorporeally. *Results*: We included 384 cases, 74 (19.3%) laparoscopic and 310 (80.7%) open right hemicolectomies. Baseline characteristics were comparable between the two groups. Conversion rate was low (2.7%). A drain was placed more often in the open colectomies (*p* < 0.001). Laparoscopic colectomies lasted longer by 25 min on average in the entire cohort (*p* = 0.002) and by 30 min in cancer-only cases without concomitant procedures (*p* < 0.001). Laparoscopic procedures yielded more lymph nodes (*p* = 0.007), as well as longer distal resection margins (*p* < 0.001) and total specimen (*p* < 0.001). There was no difference between the two approaches concerning intraoperative complications (*p* = 0.36) or need for transfusion (*p* = 0.708). There was also no difference regarding overall (*p* = 0.361) or major complications (*p* = 1), as well as anastomotic leak (*p* = 0.475), surgical site infections (*p* = 0.275) or readmission rates (*p* = 1). Hospitalisation duration was shorter by 3 days after laparoscopic surgery in the entire cohort (*p* < 0.001), as well as when cancer-only cases without concomitant procedures were considered (*p* < 0.001). *Conclusions*: Laparoscopic right hemicolectomy with intracorporeal anastomosis provides perioperative safety and pathology outcomes comparable to open surgery, while significantly reducing hospital stay.

## 1. Introduction

Colorectal cancer (CRC) remains one of the most common malignancies worldwide in both men and women, accounting for approximately 12% of all cancers, with surgical resection representing the only type of treatment with curative intent [1]. Over the last three decades, laparoscopically assisted right hemicolectomy has progressively evolved from an experimental approach to an established surgical option [2,3,4]. Its adoption for right-sided colonic resections was initially slower than for other colorectal procedures, mainly due to the extent of resection required and the complexity of right colonic vascular anatomy [2,5]. Early concerns regarding the oncological safety of laparoscopy, including reports of port-site recurrence and the theoretical risk of tumour cell dissemination due to pneumoperitoneum, were later refuted by analyses of larger series and randomised trials. Current evidence demonstrates that laparoscopic approaches provide favourable operative and postoperative outcomes [3,6,7], as well as comparable pathology outcomes in colorectal cancer surgery when compared with open approaches [3,5,7]. Despite the growing evidence supporting laparoscopic right hemicolectomy, comparative data against open right hemicolectomy in real-world practice remain important. The present study aims to evaluate intraoperative, postoperative, and pathological outcomes of laparoscopic right hemicolectomy with intracorporeal anastomosis compared with open right hemicolectomy, focusing on operative parameters, postoperative complications, recovery, and oncological specimen characteristics.

## 2. Materials and Methods

### 2.1. Patients

We retrospectively reviewed our prospectively maintained institutional database for colorectal surgery and collected data regarding right hemicolectomies performed in our department over a period of 10 years (January 2016–December 2025). Specifically, we collected data in terms of baseline patient characteristics [gender, age, ASA score, the Charlson Comorbidity Index (CCI), any previous abdominal surgery, underlying colorectal pathology, depth of infiltration (in case of cancer), presence of metastatic spread, any additional concomitant procedure, type of approach (laparoscopic or open)], operative outcomes (conversion to open surgery, intraoperative transfusion, intraoperative complications, drain placement, operative time, number of retrieved lymph nodes, distance of proximal and distal ends of the surgical specimen from the tumour, total length of surgical specimen), and postoperative outcomes (complications, reoperation, mortality, duration of hospitalisation, readmission within 30 days from discharge). As far as postoperative complications are concerned, they were graded according to the Clavien–Dindo classification [8].

We assessed the overall rate of complications, as well as the rate of major complications. We also assessed them as surgical and medical complications, and we examined certain types of surgical complications separately, such as anastomotic leak, surgical site infections and ileus. In terms of the laparoscopic technique, the colon was mobilised using the medial to lateral approach, and all the anastomoses were performed intracorporeally. All the anastomoses in both open and laparoscopic procedures were performed side to side, using a linear stapler (ECHELON FLEX 60 mm, Ethicon, Cincinnati, OH, USA or ENDOGIA 60 mm, Medtronic, Minneapolis, MN, USA). Moreover, complete mesocolic excision and central vascular ligation were routinely performed in all laparoscopic resections and in the majority of open ones from 2023 onwards. The study was conducted in accordance with the Declaration of Helsinki.

### 2.2. Statistical Analysis

Normality of data distribution was assessed using the Shapiro–Wilk test. Comparisons between two groups were performed applying the Student’s *t*-test, Welch’s *t*-test or Mann–Whitney U test for quantitative parameters, and the Chi-squared test or Fisher’s exact test for qualitative parameters, as appropriate. Multivariable linear regression analysis was applied to assess which parameters affect operative time and duration of hospitalisation. Due to the right skewness of these data, a logarithmic transformation of the dependent variables was done for the purposes of the linear regression analysis. The variance inflation (VIF) was calculated for all included parameters in the model in order to check for collinearity. Multivariable logistic regression analysis was applied to assess which parameters affect the presence of major and overall complications, as well as readmission rates. Due to the small number of events concerning major complications and readmission rates, Firth’s penalised logistic regression was applied for these outcomes. Because the percentage of missing values in the included cases was very low (<2%), we just excluded cases pairwise for both univariable and multivariable analysis. All the tests were two-tailed. The level of statistical significance was set at a *p*-value less than 0.05. The 30th edition of the Statistical Package for the Social Sciences (SPSS 30th Edition) (IBM, Armonk, NY, USA) was used for the statistical analysis. Covariates included in multivariable models were selected a priori based on clinical relevance.

## 3. Results

### 3.1. Comparison Between Laparoscopic and Open Operations—Entire Cohort

#### 3.1.1. Patient Characteristics

We included 384 cases in our analysis. There were 74 (19.3%) laparoscopic and 310 (80.7%) open right hemicolectomies. There were no statistically significant differences between laparoscopic and open operations regarding most of the baseline characteristics, namely gender [laparoscopic: male: 46/74 (62.2%), open: male: 172/310 (55.5%), *p* = 0.297], age [laparoscopic: median (IQR): 70 (19), open: median (IQR): 72 (16), *p* = 0.891], ASA score [laparoscopic: median (IQR): 2 (1), open: median (IQR): 2 (1), *p* = 0.768] and CCI [laparoscopic: median (IQR): 5 (3), open: median (IQR): 6 (3), *p* = 0.07]. Moreover, a similar portion of patients had undergone abdominal surgery in the past in the two groups [laparoscopic: 40/74 (54.1%), open: 153/310 (49.4%), *p* = 0.468]. Colon adenocarcinoma was by far the most common pathology in both groups and, although the percentage of colon cancers was somewhat higher in the open than in the laparoscopic group, there was no statistically significant difference between the two types of colectomy as far as the type of pathology is concerned [laparoscopic: adenocarcinoma: 59/74 (79.7%), adenoma: 7/74 (9.5%), Crohn’s disease: 2/74 (2.7%), other: 6/74 (8.1%), open: adenocarcinoma: 278/310 (89.7%), adenoma: 16/310 (5.2%), Crohn’s disease: 4/310 (1.3%), other: 12/310 (3.9%), *p* = 0.088]. Nonetheless, the presence of T4 tumours was more common in open than in laparoscopic procedures [laparoscopic: T4: 3/59 (5.1%), open: T4: 43/278 (15.5%), *p* = 0.035]. Furthermore, additional concomitant procedures were performed more commonly during open than during laparoscopic operations [laparoscopic: 2/74 (2.7%), open: 41/310 (13.2%), *p* = 0.01]. Finally, 12 patients in the open group presented with synchronous liver metastases and were treated with a colon-first approach due to symptoms of subacute obstruction, whereas none of the patients in the laparoscopic group had evidence of distant metastatic spread at the time of the operation. Table 1 summarises patient characteristics.

**Figure 1:**
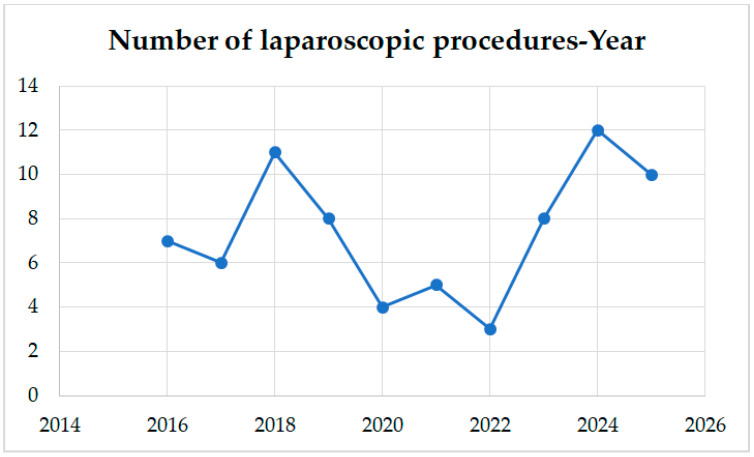
Distribution of laparoscopic operations according to calendar year.

Figure 1 depicts the distribution of laparoscopic operations according to the calendar year throughout the study period. Since the number of laparoscopic right hemicolectomies was consistently higher from 2023 onwards, we divided our cases into 2 time periods: the first one between 2016 and 2022, including 44 patients, and the second one between 2023 and 2025, including 30 patients.

#### 3.1.2. Operative Outcomes

Out of the 74 laparoscopically attempted procedures, 72 were completed laparoscopically, whereas 2 cases were converted to open, with the conversion rate being 2.7%.

There was no difference between laparoscopic and open operations concerning the need for intraoperative blood transfusion [laparoscopic: 12/74 (16.2%), open: 56/310 (18.1%), *p* = 0.708] or the rate of intraoperative complications [laparoscopic: 5/74 (6.8%), open: 13/310 (4.2%), *p* = 0.36]. A drain was placed more often in open colectomies than in laparoscopic ones [laparoscopic: 15/74 (20.3%), open: 212/310 (68.4%), *p* < 0.001]. Moreover, the duration of laparoscopic colectomies was longer by 25 min on average than open colectomies [laparoscopic: median (IQR): 145 min (40), open: median (IQR): 120 min (56), *p* = 0.002]. The longer duration of laparoscopic procedures was confirmed in the multivariable analysis (B: 0.166, 95% CI: 0.069–0.263, *p* < 0.001). The exponentiated Beta Coefficient was 1.181 (95% CI: 1.078–1.289), which means an increase in operative time by 18.1% (Table 2).

In terms of the proximal end of the surgical specimen and its distance from the tumour, there was no statistically significant difference between the laparoscopic and the open approach [laparoscopic: median (IQR): 10.9 cm (6.4), open: median (IQR): 12 cm (9), *p* = 0.24]. On the contrary, the distance of the distal end of the surgical specimen from the tumour was longer in laparoscopic colectomies, being longer by 8 cm on average when compared with open colectomies [laparoscopic: median (IQR): 20 cm (13.3), open: median (IQR): 12 cm (10), *p* < 0.001]. Similarly, when the total length of the colectomy specimen was considered, the laparoscopic specimens were longer by 6.8 cm on average than the open ones [laparoscopic: median (IQR): 36.8 cm (15.8), open: median (IQR): 30 cm (15), *p* < 0.001]. In addition, laparoscopic procedures yielded more lymph nodes than open ones [laparoscopic: median (IQR): 28 (20), open: median (IQR): 22 (12), *p* = 0.007]. When we took into account the time period, median lymph node yield was 22 (IQR: 12) and 25 (IQR: 12) for the open procedures of the first and second time period, respectively, whereas it was 24 (IQR: 22) and 30 (IQR: 11) for the laparoscopic procedures of the first and second time period, respectively. This outcome revealed a significant increase in the number of retrieved lymph nodes in the laparoscopic operations of the second time period (*p* = 0.012).

#### 3.1.3. Postoperative Outcomes

No statistically significant difference between laparoscopic and open procedures was found in terms of postoperative complication rate, either when any complication (Clavien–Dindo I–V) was considered [laparoscopic: 9/74 (12.2%), open: 51/310 (16.5%), *p* = 0.361] or when only major complications (Clavien–Dindo III–V) were considered [laparoscopic: 2/74 (2.7%), open: 10/310 (3.2%), *p* = 1]. Likewise, the minimally invasive approach resulted in similar risk of postoperative complications to the open approach in the multivariable analysis for both overall (OR: 0.759, 95% CI: 0.349–1.646, *p* = 0.485) (Table 3) and major complications (coefficient: 0.029, 95% CI: −1.652–1.332, *p* = 0.969, OR: 1.029, 95% CI: 0.192–3.789) (Table 4). There was also no difference between the two groups when either surgical [laparoscopic: 6/74 (8.1%), open: 31/310 (10%), *p* = 0.62] or medical complications [laparoscopic: 3/74 (4.1%), 20/310 (6.5%), *p* = 0.59] were examined separately.

There was no statistically significant difference between the two types of colectomies when certain types of surgical complications were examined, such as anastomotic leak [laparoscopic: 1/74 (1.4%), open: 2/310 (0.6%), *p* = 0.475], surgical site infections [laparoscopic: 2/74 (2.7%), open: 20/310 (6.5%), *p* = 0.275] and ileus [laparoscopic: 2/74 (2.7%), open: 10/310 (3.2%), *p* = 1]. Reoperation rates were low in both groups [laparoscopic: 1/74 (1.4%), open: 3/310 (1%), *p* = 0.577]. There was also 1 death in each group, which resulted in low mortality rates in both approaches [laparoscopic: 1/74 (1.4%), open: 1/310 (0.3%), *p* = 0.349]. On the other hand, the duration of hospitalisation was 3 days shorter on average after laparoscopic operations when compared with open ones [laparoscopic: median (IQR): 6 days (2), open: median (IQR): 9 (5), *p* < 0.001]. The shorter hospitalisation duration was confirmed in the multivariable analysis (B: −0.284, 95% CI: −0.374–0.193, *p* < 0.001). The exponentiated Beta Coefficient was 0.753 (95% CI: 0.688–0.824), which corresponds to a shorter hospitalisation duration by 24.7% (Table 5). Finally, readmission rates were similar between laparoscopic and open procedures [laparoscopic: 2/73 (2.7%), open: 11/309 (3.6%), *p* = 1], as it was also shown in the multivariable analysis (coefficient: −0.365, 95% CI: −2.033–0.905, *p* = 0.6, OR: 0.694, 95% CI: 0.131–2.472) (Table 6).

### 3.2. Comparison Between Laparoscopic and Open Operations—Cases with Colon Cancer Without Concomitant Procedures

#### 3.2.1. Patient Characteristics

We repeated the analysis, including only cases operated for colon cancer and excluding those with additional concomitant procedures. Thus, we ended up with 298 cases in the new analysis. There were 57 (19.1%) laparoscopic and 241 (80.9%) open right hemicolectomies. There were no statistically significant differences between laparoscopic and open operations regarding any of the baseline characteristics, namely gender [laparoscopic: male: 37/57 (64.9%), open: male: 139/241 (57.7%), *p* = 0.318], age [laparoscopic: median (IQR): 73 (18), open: median (IQR): 73 (16), *p* = 0.427], ASA score [laparoscopic: median (IQR): 2 (1), open: median (IQR): 2 (1), *p* = 0.389] and CCI [laparoscopic: median (IQR): 6 (3), open: median (IQR): 6 (2), *p* = 0.953]. In addition, a similar portion of patients had a history of previous abdominal surgery in the two groups [laparoscopic: 32/57 (56.1%), open: 116/241 (48.1%), *p* = 0.277]. Moreover, although there was a somewhat higher chance of presence of T4 tumour in the open group, this did not reach statistical significance [laparoscopic: T4: 3/57 (5.3%), open: T4: 35/241 (14.5%), *p* = 0.059]. Table 1 summarises patient and tumour characteristics of these subgroups.

#### 3.2.2. Operative Outcomes

Out of the 57 laparoscopically attempted procedures, 56 were completed laparoscopically, whereas 1 case was converted to open, with the conversion rate being 1.8%. There was no difference between laparoscopic and open colectomies in terms of the need for intraoperative transfusion [laparoscopic: 11/57 (19.3%), open: 43/241 (17.8%), *p* = 0.797] or the rate of intraoperative complications [laparoscopic: 4/57 (7%), open: 9/241 (3.7%), *p* = 0.282]. A drain was placed more often in open operations than in laparoscopic ones [laparoscopic: 6/57 (10.5%), open: 159/241 (66%), *p* < 0.001]. Furthermore, laparoscopic colectomies lasted longer by 30 min on average than open colectomies [laparoscopic: median (IQR): 150 min (40), open: median (IQR): 120 min (50), *p* < 0.001]. The difference in duration of the operation was confirmed in the multivariable analysis (B: 0.178, 95% CI: 0.09–0.266, *p* < 0.001). The exponentiated Beta Coefficient was 1.195 (95% CI: 1.094–1.305), which shows an increase in operative time by 19.5% (Table 2).

Concerning the proximal end of the surgical specimen and its distance from the tumour, there was no statistically significant difference between the laparoscopic and the open approach [laparoscopic: median (IQR): 11.3 cm (6.5), open: median (IQR): 12.5 cm (9), *p* = 0.384]. On the contrary, the distance between the distal end of the surgical specimen and the tumour was longer in laparoscopic operations, being longer by 8 cm on average when compared with open operations [laparoscopic: median (IQR): 20 (14), open: median (IQR): 12 (10), *p* < 0.001]. Likewise, the total length of the surgical specimen was longer in laparoscopic than in open colectomies by 5.8 cm on average [laparoscopic: median (IQR): 36.8 cm (16.4), open: median (IQR): 31 (15), *p* = 0.001]. In addition, laparoscopic procedures yielded more lymph nodes than open ones [laparoscopic: median (IQR): 31 (21), open: median (IQR): 22 (12), *p* < 0.001]. When we took into account the time period, median lymph node yield was 22 (IQR: 12) and 25 (IQR: 12) for the open procedures of the first and second time period respectively, whereas it was 27.5 (IQR: 21) and 32 (IQR: 13) for the laparoscopic procedures of the first and second time period, respectively. This outcome revealed a significant increase in the number of retrieved lymph nodes in the laparoscopic operations of the second time period (*p* = 0.003).

#### 3.2.3. Postoperative Outcomes

No statistically significant difference between the laparoscopic and the open approach was detected as far as postoperative complication rate is concerned, either when any complication (Clavien–Dindo I–V) was considered [laparoscopic: 7/57 (12.3%), open: 41/241 (17%), *p* = 0.382] or when only major complications (Clavien–Dindo III–V) were considered [laparoscopic: 1/57 (1.8%), open: 9/241 (3.7%), *p* = 0.693]. The similar risk of postoperative complications between laparoscopic and open colectomies was confirmed in the multivariable analysis for both overall (OR: 0.691, 95% CI: 0.29–1.65, *p* = 0.405) (Table 3) and major complications (coefficient: −0.366, 95% CI: −2.262–1.151, *p* = 0.672, OR: 0.694, 95% CI: 0.073–3.161) (Table 4). There was also no difference between the two groups when either surgical [laparoscopic: 4/57 (7%), open: 26/241 (10.8%), *p* = 0.395] or medical complications [laparoscopic: 3/57 (5.3%), open: 15/241 (6.2%), *p* = 1] were examined separately.

There was no statistically significant difference between the two types of colectomies when certain types of surgical complications were examined, such as anastomotic leak [laparoscopic: 0/57 (0%), open: 2/241 (0.8%), *p* = 1] and ileus [laparoscopic: 2/57 (3.5%), 7/241 (2.9%), *p* = 0.684]. Although surgical site infections occurred somewhat more frequently in the open than in the laparoscopic group, this difference was not statistically significant [laparoscopic: 1/57 (1.8%), open: 18/241 (7.5%), *p* = 0.139]. Reoperation rates were low in both groups [laparoscopic: 0/57 (0%), open: 3/241 (1.2%), *p* = 1]. There was also 1 death in each group, which resulted in low mortality in both approaches [laparoscopic: 1/57 (1.8%), open: 1/241 (0.4%), *p* = 0.346]. On the other hand, the duration of hospitalisation was 3 days shorter on average after laparoscopic operations when compared with open ones [laparoscopic: median (IQR): 6 (2), open: median (IQR): 9 (5), *p* < 0.001]. This was confirmed in the multivariable analysis (B: −0.293, 95% CI: −0.394–−0.192, *p* < 0.001). The exponentiated Beta Coefficient was 0.746 (95% CI: 0.674–0.825), which means a shorter hospitalisation by 25.4%. (Table 5). Finally, readmission rates were similar between laparoscopic and open procedures [laparoscopic: 1/56 (1.8%), open: 10/240 (4.2%), *p* = 0.696], and this was also shown in the multivariable analysis (coefficient: −0.724, 95% CI: −2.975–0.775, *p* = 0.38, OR: 0.485, 95% CI: 0.051–2.171) (Table 6).

## 4. Discussion

Various studies and meta-analyses have shown that laparoscopic right hemicolectomy offers significant perioperative advantages over the open approach, including faster recovery of bowel function, shorter hospital stay, reduced postoperative pain, improved cosmetic outcomes, and earlier mobilisation and feeding [3,6,7]. Intraoperatively, laparoscopy has been associated with less blood loss and equivalent length and lymph node yield, although operative time is often longer [5,6,7]. The development of intracorporeal anastomosis has further refined minimally invasive right hemicolectomy. Compared with extracorporeal anastomosis, intracorporeal anastomosis avoids excessive traction and rotation of the mesentery and bowel, allows greater flexibility in specimen extraction site, and facilitates smaller, lower abdominal incisions. These technical advantages have been associated with reduced wound complications, less postoperative pain, fewer respiratory complications, and lower incisional hernia rates [6,9,10,11]. Several studies have also reported shorter hospitalisation and at least comparable, if not reduced, rates of anastomotic leak and surgical site infection with intracorporeal anastomosis, without compromising oncological radicality [6,9,10,11]. Various technical approaches to laparoscopic right hemicolectomy have been described, including medial-to-lateral, lateral-to-medial, and cranial-to-caudal dissection. Current evidence suggests that all three approaches are safe and effective, with differences mainly relating to operative efficiency and postoperative recovery rather than pathology outcomes. However, high-quality multicentre data remain limited, and the optimal technical strategy continues to be investigated [12].

In this retrospective comparative study of right hemicolectomies, laparoscopic surgery with intracorporeal anastomosis demonstrated perioperative safety and adequacy of pathologic surrogates comparable to open surgery, while offering clear benefits in postoperative recovery [2,3,5,7]. Baseline characteristics were largely comparable between the two groups [2,3,4]. Although T4 tumours and concomitant procedures were more frequent in the open group in the entire cohort, this imbalance became less evident, without reaching statistical significance, in the cancer-only subgroup. The absence of a statistically significant difference regarding the distribution of T4 tumours between the two approaches in the cancer-only subgroup implies that there was no selection bias in terms of the primary tumour extent that affected the decision for choosing between laparoscopic and open surgery. Conversion rates were low (2.7% overall; 1.8% in cancer-only cases), indicating the feasibility of the laparoscopic approach in routine practice [3,13,14]. Intraoperative outcomes, including transfusion requirement and intraoperative complication rates, were similar between the two approaches [7,15]. Drain placement, however, was markedly more common in open surgery [16,17]. Operative time was longer in laparoscopic procedures by 25–30 min in crude analysis. After adjustment, an increase in operative time by almost 20% was detected in the laparoscopic procedures [6,11,18]. Taking into account pathology results, laparoscopic surgery yielded a significantly higher lymph node count. This finding should be interpreted as a reassuring pathological surrogate of oncologic adequacy rather than definite evidence of superior oncologic radicality, as lymph node yield may also be influenced by pathological assessment and specimen processing [5,19,20]. Proximal margins were similar, whereas distal margin length and overall specimen length were greater in the laparoscopic group [5,9,20]. Postoperative morbidity was comparable [3,5,7]. No significant differences were observed regarding overall complications, major complications, surgical or medical complications, anastomotic leak, surgical site infection, ileus, reoperation or mortality rates [5,7,15]. Length of hospital stay was consistently 3 days shorter after laparoscopy. After adjustment, a decrease in hospitalisation duration by approximately 25% was found [2,6,10]. Readmission rates were similar [21,22]. These findings were reproduced in the cancer-only subgroup, strengthening validity.

These outcomes align with large registry and propensity-matched studies comparing laparoscopic and open right hemicolectomies [2,3,4,5]. The very low conversion rate observed is at the lower end of published ranges and consistent with contemporary series, reflecting maturation of laparoscopic right hemicolectomy techniques [3,13,14]. The absence of differences in intraoperative complications and transfusion supports evidence that laparoscopic right hemicolectomy, including intracorporeal anastomosis, does not increase operative risk compared with open surgery [7,15]. The markedly reduced drain use in laparoscopy reflects modern minimally invasive practice patterns [16,17]. Operative time findings mirror the literature: laparoscopy, especially with intracorporeal anastomosis, is associated with slightly longer procedures, but differences are clinically modest and often diminish after adjustment [6,11,18]. Furthermore, higher lymph node yield in the laparoscopic group supports prior meta-analyses showing that minimally invasive right hemicolectomy achieves at least equivalent nodal harvest [5,19,20]. Longer distal and total specimen length may reflect improved visualisation and precise mesocolic dissection, consistent with studies on laparoscopic complete mesocolic excision techniques [9,20]. It should also be noted that the complete mesocolic excision technique was progressively fully adopted during the later years of the study in the laparoscopic group and in the majority of the open procedures. Postoperative morbidity equivalence aligns strongly with pooled data showing no increase in overall or major complications with laparoscopic right hemicolectomy [4,5,7]. Leak rates were low and similar, supporting evidence that intracorporeal anastomosis does not worsen anastomotic safety [5,9,11]. The most consistent benefit observed, shorter hospital stay, is one of the most reproducible advantages of laparoscopic colorectal surgery across trials and databases [2,6,10]. The absence of increased readmission rates confirms that earlier discharge does not compromise safety [21,22]. Overall, our results reinforce the current paradigm that laparoscopic right hemicolectomy with intracorporeal anastomosis preserves oncological principles while enhancing recovery, with only a modest impact on operative time.

Nevertheless, we would like to acknowledge the limitations of our study. Firstly, this is a retrospective study. Even though most of the baseline characteristics did not differ significantly between the two groups, there was no randomisation regarding the allocation of the patients to the laparoscopic or open surgery group. Furthermore, there was no regular recording in terms of the degree of postoperative pain or the exact duration and dosage of painkillers in our database, which is an outcome frequently reported in studies comparing minimally invasive with open surgical techniques. In addition, there was no recording as far as the return of bowel function is concerned, with parameters such as passing flatus, bowel movements, tolerance of oral intake or opioid consumption not having been mentioned regularly in our database. Another limitation of the present study is the absence of long-term oncologic outcomes, such as disease-free or overall survival. Therefore, conclusions regarding oncologic adequacy are limited to pathological surrogate markers. In addition, perioperative care pathways evolved during the long study period. Although a formal ERAS protocol was not systematically implemented throughout the entire study period, several ERAS principles (early mobilisation, early oral intake and multimodal analgesia) were progressively adopted in routine clinical practices. This may have contributed to the observed reduction in length of hospital stay over time. Although these constitute limitations of our study, we believe that useful conclusions can still be drawn from this retrospective comparison between laparoscopic and open right hemicolectomies.

Future research should focus on well-designed prospective randomised multicentre studies evaluating minimally invasive right hemicolectomy. In particular, further comparisons between laparoscopic and robotic approaches may help clarify potential advantages in surgical precision, ergonomics and postoperative recovery. Moreover, additional studies are required to determine the optimal intraoperative mobilisation strategy during right hemicolectomy. Although medial-to-lateral, lateral-to-medial and cranial-to-caudal approaches are all considered safe and effective, robust comparative data regarding operative efficiency, complications and oncologic outcomes remain limited, and further research is needed in order to establish which mobilisation technique should be applied.

## 5. Conclusions

Laparoscopic right hemicolectomy with intracorporeal anastomosis provides perioperative safety, postoperative morbidity and oncological quality comparable to open surgery, while significantly reducing hospital stay. Lymph node harvest and oncologically adequate resection are not compromised and may be enhanced. These findings support laparoscopic surgery with intracorporeal anastomosis as a standard approach for right-sided colonic resection in the appropriate setting.

## Figures and Tables

**Table 1:** Patient characteristics.

Parameter	Entire Cohort			Only Colon Cancer—No Concomitant Procedures		
	Laparoscopic Group (N = 74)	Open Group (N = 310)	*p*-Value	Laparoscopic Group (N = 57)	Open Group (N = 241)	*p*-Value
**Gender**						
Male	46 (62.2%)	172 (55.5%)		37 (64.9%)	139 (57.7%)	
Female	28 (37.8%)	138 (44.5%)	0.297	20 (35.1%)	102 (42.3%)	0.318
**Age**						
Mean (SD)	70.1 (12.4)	70.3 (12.1)		72.6 (11.2)	71.5 (10.9)	
Median (IQR)	70 (19)	72 (16)	0.891	73 (18)	73 (16)	0.427
**ASA score**						
Mean (SD)	2.2 (0.6)	2.2 (0.7)		2.3 (0.6)	2.2 (0.7)	
Median (IQR)	2 (1)	2 (1)	0.768	2 (1)	2 (1)	0.389
**CCI**						
Mean (SD)	5.1 (1.9)	5.7 (2)		5.6 (1.7)	5.7 (1.8)	
Median (IQR)	5 (3)	6 (3)	0.07	6 (3)	6 (2)	0.953
**Previous abdominal surgery**						
Yes	40 (54.1%)	153 (49.4%)		32 (56.1%)	116 (48.1%)	
No	34 (45.9%)	157 (50.6%)	0.468	25 (43.9%)	125 (51.9%)	0.277
**Pathology**						
Adenocarcinoma	59 (79.7%)	278 (89.7%)		57 (100%)	241 (100%)	
Adenoma	7 (9.5%)	16 (5.2%)		0 (0%)	0 (0%)	
Crohn’s disease	2 (2.7%)	4 (1.3%)		0 (0%)	0 (0%)	
Other	6 (8.1%)	12 (3.9%)	0.088	0 (0%)	0 (0%)	N/A
**T4 tumour**						
Yes	3/59 (5.1%)	43/278 (15.5%)		3 (5.3%)	35 (14.5%)	
No	56/59 (94.9%)	235/278 (84.5%)	**0.035**	54 (94.7%)	206 (85.5%)	0.059
**Concomitant procedure**						
Yes	2 (2.7%)	41 (13.2%)		0 (0%)	0 (0%)	
No	72 (97.3%)	269 (86.8%)	**0.01**	57 (100%)	241 (100%)	N/A

N/A refers to not applicable, bold values refer to statistically significant values.

**Table 2:** Multivariable linear regression analysis for operative time (minutes).

Parameter	Entire Cohort					Only Colon Cancer—No Concomitant Procedures		
	Beta Coefficient	95% CI	*p*-Value	VIF	Beta Coefficient	95% CI	*p*-Value	VIF
Gender								
(Reference: female)								
Male	0.095	0.013–0.176	**0.023**	1.195	0.059	−0.016–0.133	0.124	1.178
CCI	−0.001	−0.021–0.018	0.885	1.05	−0.008	−0.027–0.011	0.428	1.02
T4 tumour								
(reference: no)								
Yes	0.014	−0.096–0.124	0.803	1.044	−0.038	−0.141–0.066	0.474	1.042
Previous abdominal surgery								
(Reference: no)								
Yes	−0.001	−0.081–0.078	0.976	1.15	0.003	−0.07–0.075	0.945	1.151
Operation type								
(Reference: open)								
Laparoscopic	0.166	0.069–0.263	**<0.001**	1.064	0.178	0.09–0.266	**<0.001**	1.045
Concomitant procedure								
(Reference: no)								
Yes	0.316	0.195–0.436	**<0.001**	1.059	N/A	N/A	N/A	N/A
Time period								
(Reference: 1st)								
2nd	−0.221	−0.304–−0.138	**<0.001**	1.054	−0.209	−0.284–−0.133	**<0.001**	1.058

**Table 3:** Multivariable logistic regression analysis for overall postoperative complications.

Parameter	Entire Cohort			Only Colon Cancer—No Concomitant Procedures		
	OR	95% CI	*p*-Value	OR	95% CI	*p*-Value
Gender						
(Reference: female)						
Male	1.197	0.647–2.215	0.567	1.163	0.582–2.323	0.67
CCI	1.182	1.025–1.363	**0.021**	1.218	1.026–1.447	**0.025**
Previous abdominal surgery						
(Reference: no)						
Yes	0.989	0.545–1.794	0.97	1.019	0.518–2.003	0.957
Operation type						
(Reference: open)						
Laparoscopic	0.759	0.349–1.646	0.485	0.691	0.29–1.65	0.405
Concomitant procedure						
(Reference: no)						
Yes	0.956	0.391–2.336	0.921	N/A	N/A	N/A

**Table 4:** Multivariable Firth’s penalised logistic regression analysis for major postoperative complications.

Parameter	Entire Cohort			Only Colon Cancer—No Concomitant Procedures		
	Coefficient	95% CI	*p*-Value	Coefficient	95% CI	*p*-Value
Gender						
(Reference: female)						
Male	−0.07	−1.306–1.199	0.911	−0.478	−1.873–0.885	0.488
CCI	0.201	−0.086–0.476	0.166	0.221	−0.116–0.54	0.192
Previous abdominal surgery						
(Reference: no)						
Yes	−0.098	−1.323–1.108	0.872	−0.206	−1.586–1.145	0.762
Operation type						
(Reference: open)						
Laparoscopic	0.029	−1.652–1.332	0.969	−0.366	−2.62–1.151	0.672
Concomitant procedure						
(Reference: no)						
Yes	−0.126	−2.389–1.407	0.888	N/A	N/A	N/A
Time period (Reference: 1st)						
2nd	0.233	−1.042–1.38	0.702	0.077	−1.378–1.341	0.909

**Table 5:** Multivariable linear regression analysis for duration of hospitalisation (days).

Parameter	Entire Cohort				Only Colon Cancer—No Concomitant Procedures			
	Beta Coefficient	95% CI	*p*-Value	VIF	Beta Coefficient	95% CI	*p*-Value	VIF
Gender								
(Reference: female)								
Male	−0.032	−0.109–0.044	0.409	1.184	−0.049	−0.135–0.038	0.269	1.166
CCI	0.016	−0.002–0.034	0.079	1.05	0.004	−0.018–0.026	0.735	1.02
Previous abdominal surgery								
(Reference: no) Yes	−0.075	−0.15–−0.0004	**0.049**	1.147	−0.069	−0.153–0.015	0.109	1.15
Operation type								
(Reference: open) Laparoscopic	−0.284	−0.374–−0.193	**<0.001**	1.047	−0.293	−0.394–−0.192	**<0.001**	1.027
Concomitant procedure								
(Reference: no) Yes	0.133	0.02–0.247	**0.021**	1.052	N/A	N/A	N/A	N/A
Time period								
(Reference: 1st)								
2nd	−0.02	−0.097–0.058	0.619	1.034	0.006	−0.081–0.093	0.884	1.035

**Table 6:** Multivariable Firth’s penalised logistic regression analysis for readmission.

Parameter	Entire Cohort			Only Colon Cancer—No Concomitant Procedures		
	Coefficient	95% CI	*p*-Value	Coefficient	95% CI	*p*-Value
Gender						
(Reference: female)						
Male	0.045	−1.104–1.211	0.938	0.45	−0.793–1.792	0.48
CCI	−0.067	−0.368–0.219	0.655	−0.098	−0.455–0.225	0.566
Previous abdominal surgery						
(Reference: no)						
Yes	0.499	−0.642–1.707	0.391	0.388	−0.837–1.642	0.53
Operation type						
(Reference: open)						
Laparoscopic	−0.365	−2.033–0.905	0.6	−0.724	−2.975–0.775	0.38
Concomitant procedure						
(Reference: no)						
Yes	−1.214	−6.08–0.879	0.318	N/A	Ν/A	N/A
Time period						
(Reference: 1st)						
2nd	0.695	−0.427–1.792	0.217	0.805	−0.42–1.999	0.19

## Data Availability

The original contributions presented in this study are included in the article. Further inquiries can be directed to the corresponding author.
